# Excision and Circularization of Integrative Conjugative Element Tn*5253* of *Streptococcus pneumoniae*

**DOI:** 10.3389/fmicb.2018.01779

**Published:** 2018-07-31

**Authors:** Francesco Santoro, Alessandra Romeo, Gianni Pozzi, Francesco Iannelli

**Affiliations:** Laboratory of Molecular Microbiology and Biotechnology, Department of Medical Biotechnologies, University of Siena, Siena, Italy

**Keywords:** integrative conjugative element (ICE), circular form, attachment site, conjugative transposon, Tn*5253*, conjugation, mobile genetic elements

## Abstract

The integrative conjugative element (ICE) Tn*5253* of *Streptococcus pneumoniae*, conferring resistance to tetracycline and chloramphenicol, was found integrated at a 83-bp specific target site (*att*B) located in the *rbgA* gene of the pneumococcal chromosome. PCR analysis of Tn*5253*-carrying strains showed evidence of precise excision of Tn*5253* from the pneumococcal chromosome with production of (i) circular forms of the ICE in which the ends were joined by a 84-bp sequence (*att*Tn), and (ii) reconstituted chromosomal *att*B. When integrated into the chromosome, Tn*5253* was flanked by *att*L, identical to *att*B, and *att*R, identical to *att*Tn. Circular forms of Tn*5253* were present at a concentration of 3.8 × 10^-4^ copies per chromosome, whereas reconstituted *att*B sites were at 3.0 × 10^-4^ copies per chromosome. Deletion of *int-xis* of Tn*5253* abolished production of circular forms (<7.1 × 10^-6^ copies per chromosome) and was associated to the lack of Tn*5253* conjugal transfer suggesting, as expected, that Tn*5253* circular form acts as a conjugation intermediate.

## Introduction

Horizontal gene transfer, mediated by MGEs, significantly drives bacterial genome evolution including the acquisition and dissemination of new patterns of antibiotic resistance (Burrus and Waldor, 2004). Functional characterization of MGEs is essential to understand the evolution and spread of antibiotic resistance within a given bacterial species and also among different species (Frost et al., 2005). ICEs, which include CTs, are MGEs that integrate into the bacterial genome and are capable of intracellular transposition to a new genomic location or intercellular transposition to a new genome host upon conjugative transfer (Mullany et al., 2002). ICEs account for up to 25% of the genetic material in a bacterial genome (Paulsen et al., 2003) and are the major promoters of genetic diversity in bacteria (Burrus and Waldor, 2004; Johnson and Grossman, 2015).

The CT Tn*916*, carrying the *tet*(M) gene, is the prototype of the Tn*916*–Tn*1545* family of ICEs, and one of the most studied ICEs of gram positive bacteria (Santoro et al., 2014). Tn*916* was shown to excise from the bacterial chromosome producing a covalently closed circular form of the element which was called “CI.” Production of CIs of Tn*916* was demonstrated to be essential for conjugative transposition of the element (Scott et al., 1988). Recombination processes of ICEs are catalyzed by site specific recombinases (serine or tyrosine) or by DDE transposases (Ambroset et al., 2016). The Tn*916* element carries the *int* and *xis* genes which code for a tyrosine site specific recombinase and an excisionase, respectively (Lu and Churchward, 1995). Excision and circularization require both Xis and Int, whereas Int alone is sufficient for integration (Storrs et al., 1991). Dosage of Tn*916* CIs demonstrated that their number correlates with conjugation frequency and is variable among different strains (Manganelli et al., 1995).

Tn*5253* is a 64,528-bp composite ICE of *Streptococcus pneumoniae* which contains integrated two distinct genetic elements: Tn*5251*, belonging to the Tn*916*–Tn*1545* family of ICEs, and Ω*cat*(pC194) which carry *tet*(M) and *cat* resistance genes, respectively (Ayoubi et al., 1991; Provvedi et al., 1996; Santoro et al., 2010; Iannelli et al., 2014). Tn*5253* contains two pairs of *xis*/*int* recombinase genes one of which is part of Tn*5251* (Kiliç et al., 1994; Iannelli et al., 2014). Genomic sequence analysis and PCR genotyping studies demonstrated that Tn*5253*-like elements are very common in multidrug-resistant pneumococcal strains including pandemic isolates (Croucher et al., 2009; Henderson-Begg et al., 2009; Mingoia et al., 2011). A study on 240 different pneumococcal isolates of the multidrug-resistance 23F Spanish strain lineage, carrying the Tn*5253*-like element ICE*Spn*23FST81, showed that the element is maintained among all derivative strains (Croucher et al., 2011). In this work, we investigated excision and circularization of the composite ICE Tn*5253*, including the respective contribution of each *xis*/*int* recombinase pair to the conjugal transfer of the genetic element.

## Materials and Methods

### Bacterial Strains, Growth, and Mating Conditions

The bacterial strains used in this study and their relevant properties are described in **Table 1**. Bacterial growth and plate mating conjugation experiments were performed as reported (Santoro et al., 2010).

**Table 1:** *Streptococcus pneumoniae* strains.

Strain	Relevant properties^a^	Origin (Reference)
D39	type 2 Avery’s strain	Lanie et al., 2007
FP58	Conjugation recipient. *str-41*; Sm^R^ derivative of D39	Iannelli et al., 2004
Rx1	Unencapsulated derivative of D39	Pearce et al., 2002
FP10	Conjugation recipient. Δ*comC*, *str-41*; Cm^R^, Sm^R^; unencapsulated, competence deficient derivative of Rx1	Santoro et al., 2010
FP11	Conjugation recipient. Δ*comC*, *nov-1*; Cm^R^, Nov^R^; unencapsulated, competence deficient derivative of Rx1	Santoro et al., 2010
BM6001	Tn*5253* donor; *cat*, *tet*(M); original clinical strain	Dang-Van et al., 1978
DP1322	Tn*5253* donor; *cat*, *tet*(M); Cm^R^, Tc^R^; Rx1 derivative transformed with BM6001 DNA	Smith et al., 1981
FR24	Tn*5253*; *cat*, *tet*(M); Cm^R^, Tc^R^, Sm^R^; transconjugant from mating between DP1322 and FP10	This study
FR51	Tn*5253*; *cat*, *tet*(M), Δ*xis*-*int* of Tn*5253*; Cm^R^, Tc^R^, Sm^R^; Km^R^; recombinases (*orf78*-*orf79*) deletion mutant of FR24	This study
FR82	Tn*5253*; *cat*, *tet*(M), Δ*int*-*xis* of Tn*5251*; Cm^R^, Tc^R^, Sm^R^; Spe^R^; recombinases (*orf21*-*orf22*) deletion mutant of FR24	This study


*^a^str-41 indicates a point mutation conferring resistance to streptomycin, while nov-1 indicates a point mutation conferring resistance to novobiocin. Cm, chloramphenicol; Km, kanamycin; Nov, novobiocin; Sm, streptomycin; Tc, tetracycline; Spe, spectinomycin; R, resistance.*

### Pneumococcal Lysate Preparation

Pneumococcal cultures (1 ml) were harvested in exponential phase (OD_590_ about 0.2, roughly corresponding to 5 × 10^8^ CFU/ml) and centrifuged at 11,000 ×*g* for 2 min. Bacterial pellets were resuspended in 30 μl of lysis solution (DOC 0.1%, SDS 0.008%) and incubated at 37°C until clarification (about 10 min). Two hundred and seventy micro liters of TE 1×, pH 8.0 were then added to the lysate.

### PCR, Sequencing, and Sequence Analysis

PCR and direct PCR sequencing were carried out following an already described protocol (Iannelli et al., 1998; Santoro et al., 2010) and DNA sequence analysis was obtained with standard softwares. DNA sequence alignments were performed using Clustal Omega^1^ and Lalign^2^. Oligonucleotide primers and their characteristics are reported in **Table 2**.

**Table 2:** Oligonucleotide primers.

Name	Sequence (5′ to 3′)	Notes^a^	GenBank ID: nucleotides
IF327	CAA TAT AGC GTG ATG ATT GTA AT		EU351020: 1,103–1,081
IF328	AGT GAG AAT CAA ATC AGA GGT T		EU351020: 65,221–65,242
IF373	GAT GAT GAT TTG ACA CAA GAA TA		EU351020: 62,969–62,991
IF521	*ATC* *AAA CGG ATC CCC AGC TTG* TAT TCA TGT CAT CAT CCT TCC T	The first 21 nucleotides are complementary to IF149	EU351020: 63,439–63,418
IF522	*ATA TTT TAC TGG ATG AAT TGT TTT AG*T TTT GGT GTT CGC TTG GTG TTT AG	The first 26 nucleotides are complementary to IF210	EU351020: 65,260–65,283
IF523	CGG TGT ATC CAA GAT TTC CAG		EU351020: 65,862–65,842
IF517	ATT TCC TTG CGT GAT GTG TGA		EU351020: 16,810–16,830
IF681	*GTA TCG CTC TTG AAG GGA A*TA GTA CAA ATG AAT TTA CTA CTT	The first 19 nucleotides are complementary to IF101	EU351020: 17,305–17,283
IF682	*GAT CCA CTA GTT CTA GAG C*TC CCA AAT AGG AAT GTC AGT	The first 19 nucleotides are complementary to IF100	EU351020: 18,780–18,799
IF520	GTA TGG TCG TTG ATG AAG TCT		EU351020: 19,348–19,328
IF100	GCT CTA GAA CTA GTG GAT C		AY334020: 1–16
IF101	TTC CCT TCA AGA GCG ATA C		AY334020: 890–872
IF149	CAA GCT GGG GAT CCG TTT GAT		AY334018: 5–25
IF210	CTA AAA CAA TTC ATC CAG TAA AAT AT		AY334019: 880–855
IF496	GTT TGG ACA TCA TTC ATT TG		CP000410: 1,043,748–1,043,767
IF356	GAC TAG ATA GAG GCA AGC GT		CP000410: 1,043,962–1,043,943
IF138	CAG ATC AAG AAA TCA AAC TCC AA		CP000410: 725,024–725,046
IF139	CAG CAT CAT CTA CAG AAA CTC		CP000410: 725,194–725,174


*^a^Nucleotides complementary to resistance cassettes primers are reported in italics*.

### PCR Mutagenesis

Isogenic deletion mutant strains were constructed transforming FR24 with linear PCR mutagenic constructs assembled by gene splicing by overlap extension as already described (Pearce et al., 2002; Iannelli and Pozzi, 2004). Deletion of Tn*5251* *int* and *xis* CDS (*orf21* and *orf22* of Tn*5253*) was obtained with a mutagenic construct containing the *ami/aad9* spectinomycin resistance cassette flanked at the left by a 496-bp DNA fragment and at the right by a 569-bp fragment corresponding to nucleotides 16,810–17,305 and 18,780–19,348 of Tn*5253* (GenBank EU351020), respectively. The primer pair IF100/IF101 was used to amplify the spectinomycin-resistance cassette from plasmid pR412 (Bergé et al., 2002), whereas IF517/IF681 and IF520/IF682 were used to amplify the flanking fragments from FR24.

The *xis* and *int* CDSs of Tn*5253* (*orf78* and *orf79*) were deleted with a mutagenic construct containing the *ami/aphIII* kanamycin resistance cassette, flanked at the left by a 471-bp DNA fragment and at the right by a 603-bp corresponding to nucleotides 62,969–63,439 of Tn*5253* and 65,260–65,862 of Tn*5253*, respectively. The primer pair IF149/IF210 was used to amplify the kanamycin-resistance cassette from plasmid pR410 (Bergé et al., 2002), while IF373/IF521 and IF522/IF523 were used to amplify the left and right fragments from FR24. Linear PCR constructs were used directly as donor DNA in transformation experiments. Mutant strains were selected for acquisition of spectinomycin or kanamycin resistance and the correct integration of constructs was confirmed by PCR and sequencing (Iannelli and Pozzi, 2004).

### Real-Time PCR Quantification

Real-time PCR experiments were carried out with the KAPA SYBR FAST qPCR kit Master Mix Universal (2X) (Kapa Biosystems) on a LightCycler 1.5 apparatus (Roche). Real-time PCR mixture contained, in a final volume of 20 μl, 1× KAPA SYBR FAST qPCR reaction mix, 5 pmol of each primer and 1 μl of bacterial lysate as starting template. Thermal profile was an initial 3 min denaturation step at 95°C followed by 40 cycles of repeated denaturation (0 s at 95°C), annealing (20 s at 50°C), and polymerisation (10 s at 72°C). The temperature transition rate was 20°C/s in the denaturation and annealing step and 5°C/s in the polymerisation step. The primer pair IF327/IF328 amplified a 411 bp fragment used for CIs quantification, while IF496/IF356 amplified a 215 bp fragment used for free locus quantification, a 171 bp fragment of chromosomal *gyrB* gene obtained with primers IF138/IF139 was used to standardize results (**Table 2**). A standard curve for the *gyrB* gene was built plotting the threshold cycle against the number of chromosome copies using serial dilutions of chromosomal DNA with known concentration. This external standard curve was used to quantify in each sample the number of (i) chromosome copies, (ii) CIs, and (iii) reconstituted *att*B. Lower limit of detection of the assay was 10 copies/reaction. The quantification was corrected for the primer efficiency. Melting curve analysis was performed to differentiate the amplified products from primer dimers.

## Results and Discussion

### Excision of Tn*5253* From the Pneumococcal Chromosome

**FIGURE 1:**
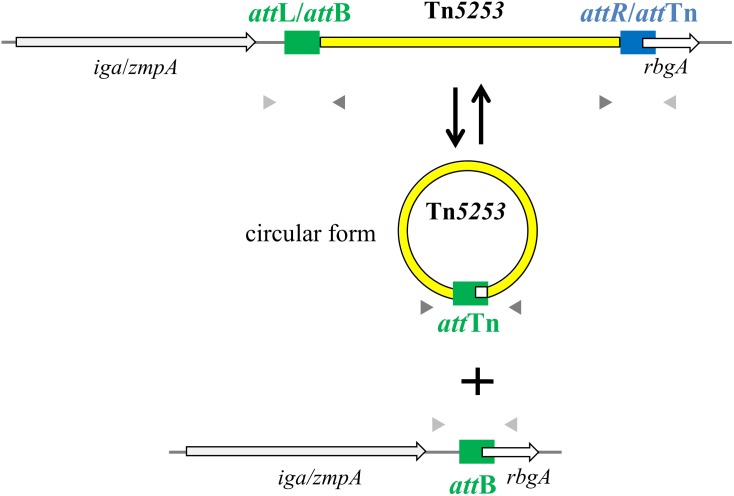
Tn*5253* excises from pneumococcal chromosome producing a circular form and a reconstitution of *att*B insertion site. In the circular form of Tn*5253* the left and right ends are joined by *att*Tn which is identical to *att*R whereas the reconstituted *att*B site is identical to *att*L. *att* sites are represented as filled rectangles, chromosomal genes as open arrows, Tn*5253* as a yellow bar. Arrowheads represent PCR primers used for circular form (dark gray) and reconstituted *att*B site (light gray) detection.

**FIGURE 2:**
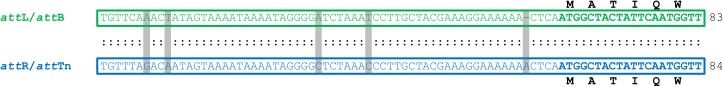
Sequence alignment of Tn*5253* attachment sites in *Streptococcus pneumoniae* D39 and derivatives. Upon integration into pneumococcal chromosome, Tn*5253* is flanked by *att*L and *att*R. The element excises from chromosome producing a circular form where the left and right ends are joined by *att*Tn and restoring the *att*B insertion site. *att*B is 83-bp long and is identical to *att*L while *att*Tn is 84-bp long and is identical to *att*R. *att*R-*att*Tn contain 4 nucleotide changes and 1 insertion compared to *att*L-*att*B. *att*L-*att*B contains the first 20 nucleotides of *rbgA* CDS, whose deduced amino acid sequence is reported, together with the 63 nucleotides upstream of the start codon. Within the sequences, identical nucleotides are indicated by colon, changes are shaded. Amino acids are indicated using one-letter code abbreviations. Lengths of attachment sites are reported on the right.

PCR analysis of cell lysates of Tn*5253*-carrying pneumococcal strains showed evidence of precise excision of Tn*5253* from its specific attachment site (*att*B) in the pneumococcal chromosome. This excision was investigated in liquid cultures of BM6001, the clinical isolate in which Tn*5253* was originally found, and four other Tn*5253*-carrying laboratory strains all deriving from classic type 2 D39 (**Table 1**). Using divergent primers (IF327, IF328; **Table 2**) designed on the ends of the element, PCR analysis showed the presence of junctions between the left and right ends of Tn*5253* (*att*Tn), whereas with convergent primers (IF496, IF356; **Table 2**) designed on the regions flanking the insertion site it was possible to show the presence of chromosomes with reconstituted target sites for integration of Tn*5253* (*att*B) (**Figure 1**). In all Tn*5253*-carrying pneumococci, DNA sequence analysis of PCR fragments indicated that: (i) *att*Tn was 84 bp in size and was identical to *att*R, the 84-bp direct repeat present at the right end of the integrated element, whereas (ii) *att*B was 83 bp in size and was identical to *att*L, the direct repeat present at the left end of the integrated element (**Figure 2**). The *att*R-*att*Tn repeat contained 4 nucleotide changes and 1 insertion compared to *att*L-*att*B (**Figure 2**). These results suggest that in Tn*5253*-carrying strains, recombination occurs between the two imperfect direct repeats *att*L and *att*R leading to precise excision of the element from the chromosome, with production of circular forms of Tn*5253* in which the ends are joined by *att*R/*att*Tn, while the *att*L/*att*B repeat remains in the bacterial chromosome (**Figure 1**).

### Quantification of Circular Forms and Reconstituted *att*B Sites

To obtain a quantitative estimate of Tn*5253* excision from the *S. pneumoniae* chromosome, Real-time PCR was used to quantify concentration of circular forms and reconstituted *att*B sites in liquid bacterial cultures. Different Tn*5253*-carrying laboratory strains of D39 ancestry, and BM6001 showed very homogeneous quantitative results (**Table 3**). In the laboratory strain DP1322 circular forms of Tn*5253* were present at a concentration of 5.1 × 10^-4^ (±2.7 × 10^-4^) copies per chromosome, whereas reconstituted *att*B sites were at 2.1 × 10^-4^ (±3.0 × 10^-5^) copies per chromosome. These values were comparable to those obtained in the Tn*5253*-carrying laboratory strains (**Table 3**). Autonomous plasmid-like replication is common in ICEs and contributes to the stability and maintenance of these elements (Lee et al., 2010; Carraro et al., 2015; Johnson and Grossman, 2015). The hypothesis that also Tn*5253* circular forms undergo few cycles of autonomous replication can explain why the copy number of circular forms is higher than the copy number of the reconstituted *att*B site.

**Table 3:** Real-time PCR quantification of Tn*5253* circular form and reconstituted *att*B^a^.

Strain	Circular forms	Reconstituted *att*B sites	Conjugation frequency^b^
BM6001	1.8 × 10^-4^ (±1.5 × 10^-5^)	2.4 × 10^-4^ (±3.6 × 10^-5^)	3.4 × 10^-7^
DP1322	5.1 × 10^-4^ (±2.7 × 10^-4^)	2.1 × 10^-4^ (±3.0 × 10^-5^)	1.6 × 10^-4^
FR24	5.4 × 10^-4^ (±3.1 × 10^-4^)	3.7 × 10^-4^ (±3.3 × 10^-5^)	2.0 × 10^-4^
FR51	none (≤7.1 × 10^-6^)	none (≤7.1 × 10^-6^)	none (<9.9 × 10^-8^)


*^*a*^Concentration was expressed as copies per chromosome. ^*b*^Frequency refers to mating experiments where *S. pneumoniae* FP11 was the recipient.*

### Site-Specific Integration of Tn*5253* Into the *rbg*A Gene

In *S. pneumoniae* D39 and in its derivatives used as conjugation recipients (**Table 1**) DNA sequence analysis showed that *att*B of Tn*5253* was 83 bp in size, and was always located within the *rbgA* gene (nucleotides 1,043,779 to 1,043,861, GenBank CP000410) (**Figure 1**). The ribosomal biogenesis GTPase A encoded by *rbgA* is a conserved, essential bacterial protein involved in the 50S ribosome subunit assembly (Uicker et al., 2006). The *att*B site contained the first 20 nucleotides of the *rbgA* CDS together with the 63 nucleotides upstream of the start codon (**Figures 1, 2**). The junction fragments of Tn*5253* with the bacterial chromosome were investigated in a total of 12 transconjugants obtained in independent matings in which Tn*5253* was transferred by conjugation from 2 pneumococcal donors (BM6001 and DP1322; **Table 1**) to 3 pneumococcal recipients (FP58, FP10, FP11; **Table 1**). Left and right junction fragments were amplified by PCR (using primer pairs IF496/IF327 and IF328/IF356) and sequenced in all transconjugants. DNA sequence analysis showed that in all cases Tn*5253* integration occurred at the same site within *rbgA*, between the two direct repeats: (i) *att*L, corresponding to the *att*B of the recipient and (ii) *att*R, corresponding to the *att*Tn of the circular forms of Tn*5253* (**Figure 2**). These results indicated that *att*R, one of the two repeats flanking Tn*5253* in the donor chromosome, was always transferred by conjugation to the recipients. Since the integrated form of Tn*5253* was invariably flanked by *att*B at the left end (*att*L) and *att*Tn at the right end (*att*R), we hypothesize a polarization in the DNA integration process. Site specific integration of MGEs often occurs at one end of essential and highly conserved genes, such as the 3′ end of tRNA genes and the 3′ or 5′ end of genes coding for ribosomal proteins (Ambroset et al., 2016). Also for Tn*5253* integration occurs at the 5′ end of an essential gene, with target site duplication allowing restoration of an intact CDS. The use of essential and conserved genes as target sites guarantees the presence and conservation of *att*B in bacterial genomes favoring the spread of ICEs such as Tn*5253*, which can overpass the border of a single species and thus favor the dissemination of multiple antibiotic resistance genes. In fact, in other bacterial species such as *Streptococcus pyogenes* and *Streptococcus mitis*, Tn*5253*-like elements are found integrated at the 5′ end of *rbgA* orthologous genes (Mingoia et al., 2014; Petrosyan et al., 2016).

### Recombinase Genes Involved in Tn*5253* Excision

**FIGURE 3:**
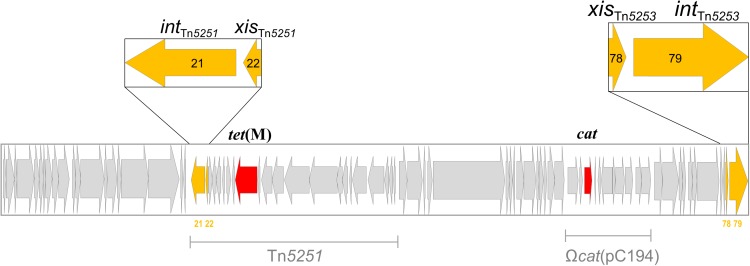
The composite integrative conjugative element (ICE) Tn*5253* contains two distinct genetic elements integrated: (i) Tn*5251* which carries *tet*(M) and is able to transfer by conjugation autonomously; (ii) Ω*cat*(pC194) which contains *cat,* is not conjugative, but is capable of intracellular transposition. Two sets of *xis*/*int* recombinase genes are carried by Tn*5253*, one set (*orf78/orf79*) is at the right end of the element, while the other (*orf21*/*orf22*) belongs to Tn*5251*. ORFs and their transcription direction are indicated as arrows, sequences corresponding to Tn*5251* and Ω*cat*(pC194) are indicated by solid bars.

Two sets of *xis*/*int* recombinase genes are present in the sequence of Tn*5253*, one set (*orf78/orf79*) is at the right end of the element,while the other (*orf21*/*orf22*) belongs to Tn*5251* (**Figure 3**). Excisionase Xis and tyrosine integrase Int are known to work in synergy, for this reason we decided to construct mutants where the *xis* and *int* genes were both deleted. For each set of *xis*/*int* recombinase genes, we constructed an isogenic deletion mutant in the Tn*5253*-carrying strain FR24. In FR51 a 1,820-bp DNA fragment (position 63,440–65,259, GenBank No. EU351020) encompassing *orf78/orf79* CDSs was deleted and replaced with the 876-bp *ami/aphIII* cassette. In FR82 a 1,474-bp DNA fragment (position 17,306–18,779, GenBank No. EU351020) encompassing *orf21/orf22* CDS was deleted and replaced with the 894-bp *ami/aad9* cassette (**Table 2**). The deletion of *xis*/*int* of Tn*5251* abolished the production of circular forms and the conjugal transfer of Tn*5251*, but did not affect the frequencies of Tn*5253* circular forms, of *att*B site reconstitution, and of Tn*5253* conjugal transfer (data not shown). Deletion of *xis*/*int* of Tn*5253* in FR51 abolished the circular forms generation and the reconstitution of *att*B site (<7.1 × 10^-6^ copies per chromosome for both genetic structures, **Table 3**). The absence of circular forms in FR51 was associated to the lack of Tn*5253* conjugal transfer suggesting, that the circular form of Tn*5253* acts as a conjugation intermediate as proposed for other characterized ICEs including Tn*916*. Data obtained using *xis*/*int* deletion mutants showed that the two recombinase pairs act independently and do not complement each other. This finding suggests that the association between the two elements is physical but not functional.

## Conclusion

In this work we have shown that: (i) Tn*5253* is capable of precise excision from the chromosome, producing circular forms of the element, and leaving chromosomes with reconstituted *att*B sites; (ii) in the circular forms, the two ends of Tn*5253* are joined by *att*Tn, an 84-bp DNA fragment identical to the *att*R junction fragment flanking the element in its integrated form; (iii) *att*R is always transferred to the recipient strain during conjugation; (iv) production of Tn*5253* circular forms and their conjugal transfer were abolished when *xis*/*int* of Tn*5253* were deleted. Even if the importance of ICEs in shaping bacterial genomes is widely recognized and nucleotide sequences of ICEs are increasingly available, a functional characterization is available only for a few of these genetic elements. This work on Tn*5253* contributes to elucidating the transfer functions of one of the prototypes of ICEs of gram positive bacteria.

## Author Contributions

FS, FI, and GP designed the experiments. FS and AR performed the experimental work. All authors analyzed and interpreted the data. FI, FS, and GP wrote the paper.

## Conflict of Interest Statement

The authors declare that the research was conducted in the absence of any commercial or financial relationships that could be construed as a potential conflict of interest.

## Footnotes

1 https://www.ebi.ac.uk/Tools/msa/clustalo/

2 http://vega.igh.cnrs.fr/bin/lalign-guess.cgi

## Abbreviations

CI: circular intermediate
CDS: coding sequence
CT: conjugative transposon
HGT: horizontal gene transfer
ICE: integrative conjugative element
MGE: mobile genetic element
